# T Cells Contribute to Pathological Responses in the Non-Targeted Rat Heart following Irradiation of the Kidneys

**DOI:** 10.3390/toxics10120797

**Published:** 2022-12-18

**Authors:** Marek Lenarczyk, Ammar J. Alsheikh, Eric P. Cohen, Dörthe Schaue, Amy Kronenberg, Aron Geurts, Slade Klawikowski, David Mattson, John E. Baker

**Affiliations:** 1 Radiation Biosciences, Medical College of Wisconsin, Milwaukee, WI 53226, USA; mlenarczyk@mcw.edu; 2 Department of Physiology, Medical College of Wisconsin, Milwaukee, WI 53226, USA; ammaralsheik@gmail.com (A.J.A.); ageurts@mcw.edu (A.G.); 3 Department of Medicine, Division of Nephrology, New York University, New York, NY 10016, USA; eric.cohen@nyulangone.org; 4 Department of Radiation Oncology, University of California at Los Angeles, Los Angeles, CA 90095, USA; dschaue@mednet.ucla.edu; 5 Biological Systems and Engineering, Lawrence Berkeley National Laboratory, Berkeley, CA 94720, USA; a_kronenberg@lbl.gov; 6 Department of Radiation Oncology, Medical College of Wisconsin, Milwaukee, WI 53226, USA; sklawikowski@mcw.edu; 7 Department of Physiology, Medical College of Georgia, Augusta, GA 30912, USA; dmattson@augusta.edu

**Keywords:** keyword radiation, kidney, heart, immune system, T cell knock down, rat, nephropathy, cardiac fibrosis

## Abstract

Heart disease is a significant adverse event caused by radiotherapy for some cancers. Identifying the origins of radiogenic heart disease will allow therapies to be developed. Previous studies showed non-targeted effects manifest as fibrosis in the non-irradiated heart after 120 days following targeted X-irradiation of the kidneys with 10 Gy in WAG/RijCmcr rats. To demonstrate the involvement of T cells in driving pathophysiological responses in the out-of-field heart, and to characterize the timing of immune cell engagement, we created and validated a T cell knock downrat on the WAG genetic backgrou nd. Irradiation of the kidneys with 10 Gy of X-rays in wild-type rats resulted in infiltration of T cells, natural killer cells, and macrophages after 120 days, and none of these after 40 days, suggesting immune cell engagement is a late response. The radiation nephropathy and cardiac fibrosis that resulted in these animals after 120 days was significantly decreased in irradiated T cell depleted rats. We conclude that T cells function as an effector cell in communicating signals from the irradiated kidneys which cause pathologic remodeling of non-targeted heart.

## 1. Introduction

Heart disease is a significant detrimental event caused by radiation therapy for some cancers [1]. The pathogenesis of radiogenic heart disease is not well defined since the heart is not always directly in the irradiated field. Identifying the origins of radiogenic heart disease will enable better therapies to be developed. Irradiation of the organs below, but not above, the diaphragm with 10 Gy of X-rays in wild-type WAG/RijCmcr (WAG) rats causes fibrotic heart disease that is quantitatively and qualitatively similar to that seen following total body irradiation [2]. These findings suggest local radiation can exert a systemic response to cause non-targeted, i.e., abscopal effects. Non-targeted effects are effects manifest in non-irradiated cells that received signals communicated from an irradiated cell [3].

Targeted irradiation of the kidneys with 10 Gy in wild-type WAG rats causes mature T cells, macrophages and natural killer cells to infiltrate the cortex and medulla 120 days after exposure [4], though the response of the immune system at an earlier time point remained undefined. Both human [5,6] and rat [4] kidneys contain myeloid and lymphoid cells that may respond to local damage from radiation and thereby determine whether these insults are resolved or progress and reach beyond the irradiated organ. 

To examine the role of T cells in the pathogenesis of radiation nephropathy, we initially chose a genetic model of immune cell deficiency that lacks functional T cells using the Dahl S rat [7]. In a local kidney irradiation model, we have shown significantly less renal radiation injury of genetically T cell knock down Dahl S rats [4]. This supports a role for the immune system in mediating radiation nephropathy. This finding raised the untested possibility that T cells may also be responsible for cardiac remodeling. However, targeted irradiation of the kidneys with 10 Gy in T cell knock down Dahl S rats did not result in less fibrosis in the non-targeted heart. The high level of cardiac fibrosis in sham-irradiated wild-type and T cell knock down Dahl S rats likely obscured any additional effects of radiation [4]. To overcome this limitation, we adapted a model of genetic depletion of T cells to WAG rats, a strain of rat susceptible to non-targeted cardiac injury, to determine whether T cells are involved in communicating signals between the irradiated kidney and the non-targeted heart.

The objectives of our study were to (i) determine the timing and extent of immune cell infiltration in kidney and heart after targeted irradiation of kidneys (ii) generate and validate a T cell knock down rat on the WAG genetic background and (iii) determine the role of T cells on nephropathy caused by targeted irradiation of the kidneys as well as development of pathology in the non-targeted heart. The principal findings were that immune cell engagement following kidney irradiation is time dependent, and that T cells function as effector cells in communicating a signal from the irradiated kidneys that causes pathologic remodeling of non-targeted heart. 

## 2. Materials and Methods

### 2.1. Compliance with Design and Statistical Analysis Requirements

Group sizes were equal. Eight rats were included in each experimental group. The numbers in each experimental group were based on our previous experiences with the same measurements in comparable studies. Animals were allocated at random to each group. The identity of the animals in each experimental group was known to the investigator responsible for initiating and continuing with an intervention, e.g., radiation. The identity of the animal under study was unknown to the investigator performing the experimental measurement or the analysis. These investigators were different persons. The study tested the hypothesis that local targeted irradiation of the kidneys causes pathology in the non-targeted heart through activation of T cells.

### 2.2. Experimental Animals

Male wild-type WAG and WAG^CD247-/-^ rats with a null deletion in *Cd247* (CD3 ζ) were used for these studies that started at 5–6 weeks of age. This age corresponds to childhood in humans [8]. Animals were housed in pairs and maintained on TEKLAD 8904 rat food and water ad libitum and provided with wood chip bedding (Sani-Chips^®^) during the study at the Biomedical Resource Center of the Medical College of Wisconsin, Milwaukee, WI, USA. The rats were kept on a 12 h light, 12 h dark cycle. The cages were maintained at a temperature of 20 ± 1 °C and relative humidity of 50–80%. The drinking water distributed to the rats was additionally purified by reverse osmosis and then chlorinated. The rats drank water that was not acidified. The start time of the study was defined as the moment rats were irradiated or sham irradiated.

### 2.3. Generation of T Cell Knock down Rat on the WAG/RijCmcr Genetic Background

CRISPR/Cas9 mutagenesis was used to knock down *Cd247* in the wild-type WAG strain. *Cd247* encodes the CD3 ζ chain that is involved in the assembly and expression of the T-cell receptor complex. A single guide RNA (sgRNA) targeting the *Cd247* exon 2 sequence GATGGAATCCTCTTCATCTACGG (protospacer adjacent motif in bold) was injected along with SpCas9 protein into one-cell WAG embryos. A founder offspring harboring a 17-bp frameshift indel mutation deleting GAATCCTCTTCATCTAC (rn6.0 chr13:84,064,185-84,064,201) was identified and confirmed by Sanger sequencing. The frameshift mutation in this strain (WAG-*Cd247*^em9Mcwi^; RGDID: 152985692) was predicted to cause a premature truncation of the normal 165 amino acid protein coding sequence after only 37 amino acids, lacking most of the transmembrane and the entirety of the external cellular protein domains. This founder was back-crossed to the WAG/RijCmcr strain to establish a mutant breeding colony, and offspring were genotyped using gene specific primers 5′-CAGTTTGTGGTCTGTGTGAAGTG-3′ and 5′-GCACAGCTGAAGAGTCTGAGAG-3′.

### 2.4. Lateral Irradiation

**Figure 1:**
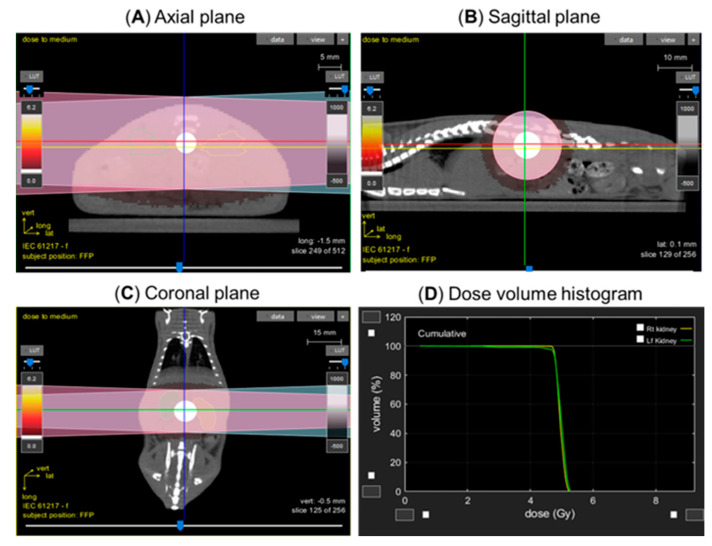
Image-guided localized kidney radiation technique. Computed tomography images of a representative male rat at 5–6 weeks of age with a 1.5-cm-diameter collimator plan of 5 Gy encompassing both kidneys in equally weighted beams (two laterals) are shown in the axial (**A**), sagittal (**B**), and coronal (**C**) planes. The kidneys are located within the central circle of each image. (**D**) dose volume histogram demonstrating dose to the kidneys.

To test whether local irradiation of both kidneys resulted in pathology to the non-targeted heart in wild-type and WAG^CD247-/-^ rats, a lateral field was used to avoid irradiation of the liver and intestines. Rats were anesthetized using 3% isoflurane/air during irradiation. The adrenals were also exposed to radiation. Animals were positioned in the prone position and oriented in the beam line with their sides at right angles to the beam to ensure consistent irradiation throughout the rat. The anatomic location of the kidneys was confirmed using a PaxScan 2520V Amorphous Silicon Digital X-ray Imager (Varian) attached to the X-RAD SmART irradiator unit to capture images of the exposed field (Figure 1). Rats (*n* = 8/group) received local kidney irradiation with a total of 10 Gy resulting from two dose fractions of 5 Gy, separated by minutes, with the rat rotated once 180 degrees. Sham irradiated rats served as controls. The dose rate for local kidney irradiation studies was 4.237 Gy/min. The X-ray machine was operated at 225 kVp, 5 mA, and 0.3 mm Cu filter, using a 1.5 cm circular collimator that encompassed both kidneys. Images of a typical rat with the radiation strategy and dose-volume histogram summary are provided in Figure 1.

### 2.5. Irradiator and Dosimetry

Local kidney irradiation was accomplished using the high-precision image-guided X-RAD SmART irradiator (Precision X-Ray, North Branford, CT, USA) between 8:00 a.m. and 10:00 a.m. The output of the irradiator was checked regularly using a calibrated ionization chamber

### 2.6. Kidney Injury Measurement

Blood was withdrawn from the jugular vein by venipuncture from wild-type and WAG^CD247-/-^ rats at 20-day intervals beginning 40 days post-exposure and up to 120 days after local kidney irradiation, and from control sham irradiated rats. Serum was evaluated for blood urea nitrogen (BUN) levels (Wisconsin Diagnostic Laboratories, Milwaukee, WI, USA).

### 2.7. Immune Cell Isolation and Flow Cytometry

Immune cells were isolated and flow cytometry was performed as previously described [9,10]. Peripheral blood mononuclear cells (PBMCs) were isolated from heparinized whole blood over Histopaque-1083 (Cat# 10831, Sigma-Aldrich, Saint Louis, MO, USA). Approximately 1 × 10^ 6^ cells were stained with an antibody cocktail containing anti-CD45 PE-Cy7 (BioLegend, catalog no. 202214, clone OX-1, 0.062 µg, San Jose, CA, USA), or anti-CD3 PerCP eFluor710 (eBioscience, catalog no. 46-0030-82, clone G4.18, 0.062 µg), or anti-CD8a FITC (BioLegend, catalog no. 201703, clone OX-8, 0.125 µg), or anti-CD4 APC-Cy7 (BioLegend, catalog no. 201518, clone W3/25, 0.062 µg), or anti-CD11b/c Alexa eFluor 660 (eBioscience, catalog no. 50-0110-82, clone OX-42, 0.062 µg), or anti-CD45R PE (BD Bioscience, catalog no. 554881, clone HIS24, 0.125 µg) in 100 µL of wash buffer [PBS (1×) without Ca^2+^ and Mg^2+^, 2% FBS, and 2 mM EDTA]. Excess antibody was washed out and cells then resuspended in 300 µL of wash buffer. DAPI was added for quantification of dead cells 10 min before samples were run on an LSR II flow cytometer (BD Biosciences, San Jose, CA, USA) acquired and analyzed with FlowJo Software (FlowJo, Ashland, OR, USA).

### 2.8. Histology

To evaluate tissue damage at 20 days, 40 days and 120 days after irradiation, the entire heart and both kidneys (*n* = 6/group) were removed from anesthetized rats. Tissues were then fixed by immersion in 10% formalin (*v/v*) as described previously [2]. Prior to embedding in paraffin wax for histology, the heart was oriented between the base and the apex, and the kidneys positioned along the mid-coronal plane. The fixed tissue samples were embedded; kidney samples in coronal orientation and heart samples in the transverse plane. Sections 4 µm thick were stained with Masson’s-trichrome. Traverse sections of heart tissue were always taken from the center of each ventricle from each animal. For the kidney, longitudinal segments were obtained for cortex and medulla. Ten sections of the entire heart and kidney were utilized for morphometric analysis as described previously [2]. Sections of the heart (*n* = 3–4/group) were analyzed in a blinded fashion. All selected images of the heart microvasculature were acquired using a Nikon E-55i microscope equipped with a Nikon DS-Fi1 camera. The collagen content was measured in the areas of perivascular and wall space on calibrated images (25–35/rat/group) by planimetry and quantified using MetaMorph image analysis program version 7.7.0.0. (Molecular Devices, Downington, PA, USA). Planimetry determined the true area of the coronary vessel, thereby eliminating errors that may have been caused by vessel compression and collapse. The collagen content of the coronary vessels was defined as the total area stained blue with trichrome, expressed as a percentage of the luminal area and then averaged per animal and grouped by treatment for statistical analysis. Perivascular cardiac fibrosis was defined as an increase in collagen content in irradiated animals above the value for sham-irradiated animals [2]. Renal collagen content was measured over the area of entire kidney sections stained blue with trichrome and expressed as a percentage of the total area of the kidney section. 

### 2.9. Immunohistochemistry

Paraffin-embedded sections were run on a DAKO Autostainer Plus automated staining platform. Antibodies included T cells with DAKO Rabbit Polyclonal CD3^+^ (A0452, 1:100), B cells with Cell Signaling Rabbit Monoclonal CD20^+^ (E3N70, 1:600), natural killer cells with Cell Marque mouse monoclonal CD56^+^ (156R-94, 1:200), and macrophages with EMB Millipore mouse monoclonal CD68^+^ (MAB1435, 1:100). Preliminary treatment t was performed using citrate buffer for CD3^+^, CD20^+^, CD56^+^ and CD68^+^, using EDTA recovery. The standard Labeled Streptavidin Biotin method was used for detection with all antibodies. Primary antibodies were incubated for 1 h and biotinylated secondary antibodies were incubated for 30 min at ambient temperature (anti-mouse Jackson Immuno 715-066-151 and anti-rabbit Jackson Immuno 715-066-152) followed by a 15 min period of incubation with streptavidin-HRP (DAKO P039701-2). Visualization was accomplished with DAB+ application (DAKO DAB+K346811-2). All slides were counterstained with Modified Mayer’s Hematoxylin (DAKO S3309330-2) and blued with 0.1% ammonium water and mounted with synthetic mounting media. Exclusion of the primary antibody acted as a negative control. Antibodies were analyzed and authenticated for immunohistochemistry (IHC) using the Histology Core Facility at Children’s Wisconsin [11]. The IHC images were then digitally recorded using a high-resolution, whole slide scanner (NanoZoomer HT 2.0, Hamamatsu, Japan) at 40× magnification and the data were reviewed using NDPview (version 2.7.43, Hamamatsu, Japan) for virtual image exploration. Scanned images were then imported into the Image Analysis Software (Visiopharm, Hørsholm, Denmark) and quantified at 20× magnification for DAB expression. The software was programmed to capture DAB-positive (CD marker) and kidney tissue areas using a preset threshold and linear Bayesian classification. The processed image was pseudo color coded for DAB-positive CD areas and tissue areas for each kidney section. The data was then expressed as a percentage value of CD+ area per total kidney tissue area. Data were collected for each animal and grouped per CD marker. Finally, the statistical analysis was performed to show the difference between sham and irradiated CD+ groups. IHC images were recorded and evaluated at the Imaging Core of Children’s Research Institute at the Medical College of Wisconsin.

### 2.10. Statistical Analysis

All numerical values were expressed as the mean ± standard deviation (SD) or standard error of the mean (SEM) as indicated. All statistical analyses were performed using Sigma Plot^®^ 11.0 software. Exploratory data analysis used a Shapiro–Wilk test [12] followed by unpaired student’s t test to perform 2-group comparisons. Data failing the Shapiro–Wilk test of normality [12] were analyzed by the Mann–Whitney rank sum (U) test [13]. The customary threshold for statistical significance (*p* < 0.05) was used in the analysis. Individual *p* values at specific time points within the longitudinal studies are reported in the figures.

## 3. Results

### 3.1. Immune Cell Infiltration in Kidney after Targeted Irradiation Is a Late Response

**Figure 2:**
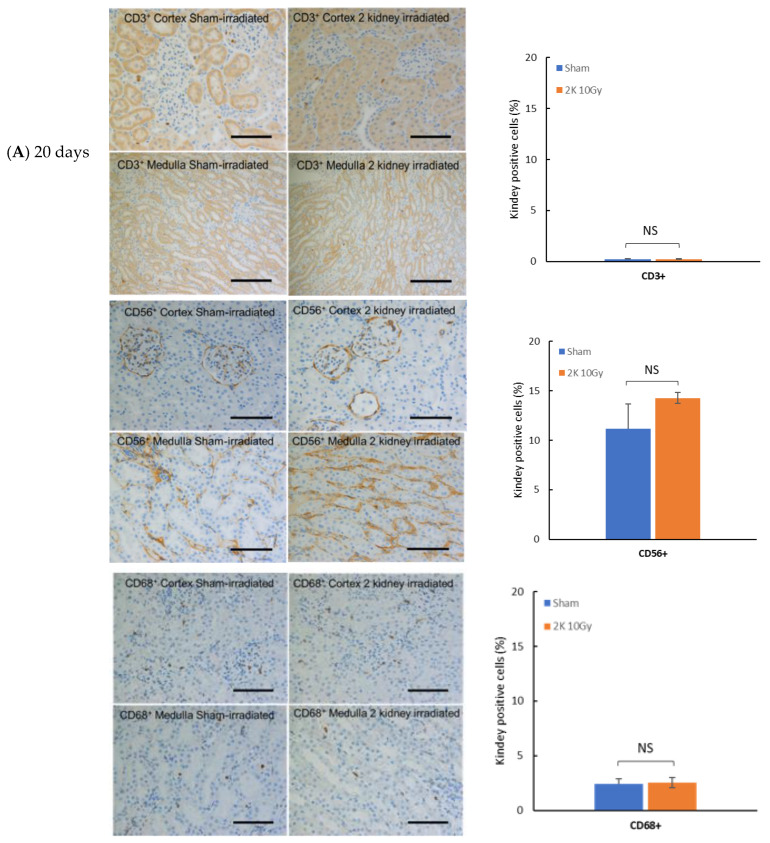

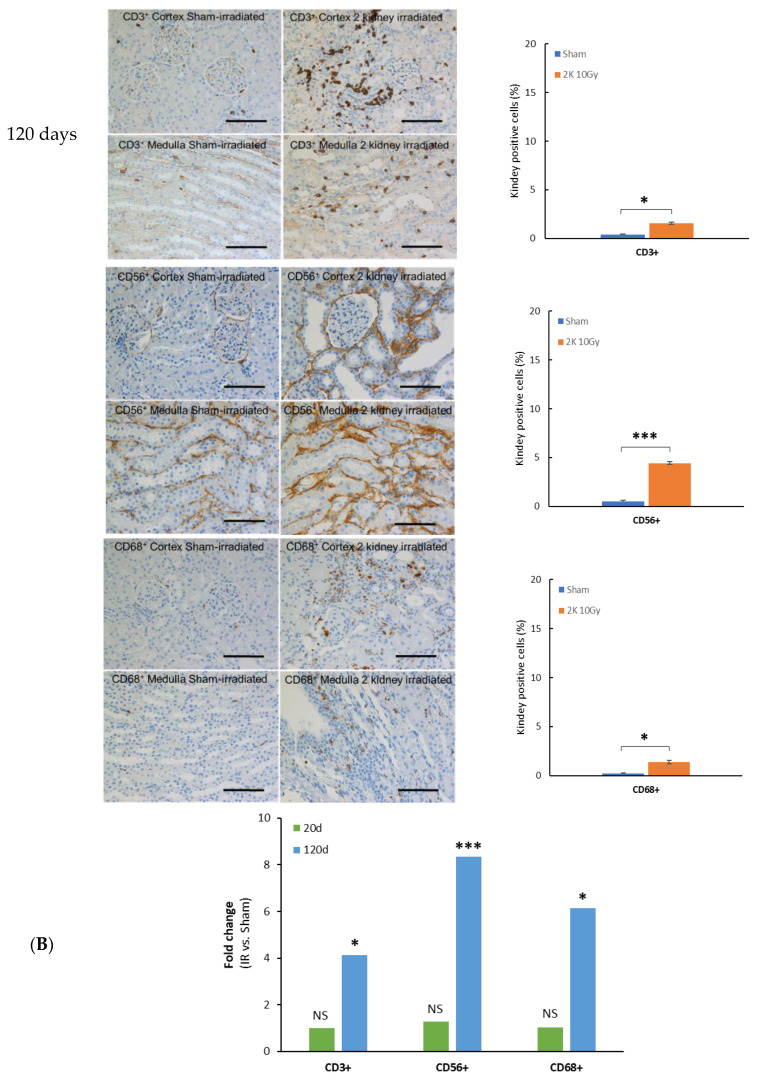
Immune cell infiltration of the cortex and medulla of kidney after local X-irradiation with 10 Gy in wild-type rat. (**A**) T cells (CD3^+^), natural killer cells (CD56^+^) and macrophages (CD68^+^) appear as brown color. The horizontal scale bar represents 100 microns. Images are representative data from 3–4 animals per group. Quantification of immune cells in cortex and medulla of kidney. (**B**) Comparison of changes between 20 days and 120 days. Data are mean + SEM. * = *p* < 0.05 vs. sham-irradiated control. *** = *p* < 0.001 vs. sham-irradiated control.

To examine the timing of immune cell engagement after local kidney irradiation, IHC was performed on kidney tissue from wild-type WAG rats at 20 and 120 days that were sham-irradiated or irradiated with 10 Gy. Immune cells were present in sham-irradiated kidneys at 20 and 120 days after the start of the study with T lymphocytes (CD3^+^), natural killer cells (CD56^+^), and macrophages (CD68^+^) detectable in the cortex and medulla (Figure 2). There was no increase in these immune cells in the kidney at 20 days after irradiation (Figure 2), a time before radiation nephropathy as measured by increased BUN is present [2]. In contrast at 120 days, there is a significant increase in CD3^+^, CD56^+^ and CD68^+^ positive cells in the cortex and medulla regions of the kidney (Figure 2); a time when radiation nephropathy is always evident histologically [2]. The extent of immune cells present in the kidney before and after irradiation was greatest for natural killer cells, spatially associated with the medulla (Figure 2). In sham-irradiated kidneys, there was a decrease in CD56^+^ and CD68^+^ positive cells from 20 to 120 days. 

### 3.2. Immune Cells Do Not Infiltrate the Heart after Kidney Irradiation

**Figure 3:**
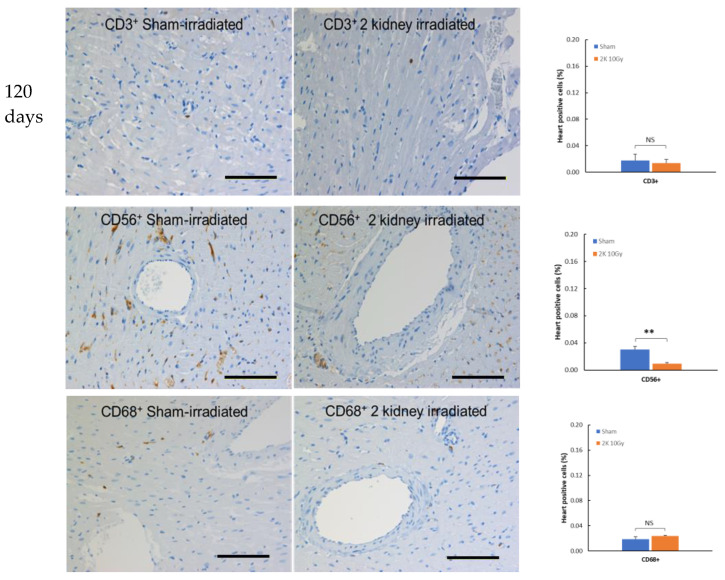
Immune cells in heart 120 days after local irradiation of kidneys with 10 Gy of X-rays in wild-type rat. T cells (CD3^+^), natural killer cells (CD56^+^), and macrophages (CD68^+^) appear as brown color. The horizontal scale bar represents 100 microns. Images are representative data from 3–4 animals per group. Quantification of immune cells in heart. Data are mean + SEM. ** = *p* < 0.01 vs. sham-irradiated control.

To determine if local irradiation of the kidneys engages the immune system outside the targeted irradiation volume, IHC was performed on non-targeted heart tissue from wild-type WAG rats at 120 days after sham or 10 Gy irradiation, a time when radiogenic fibrosis is present [4]. Immune cells were resident in sham-irradiated hearts 120 days after the start of the study with CD3^+^, CD56^+^, and CD68^+^ positive cells detectable (Figure 3), although at lower levels than in kidney (Figure 2). There was no increase in CD3^+^ or CD68^+^ positive cells in heart 120 days following kidney irradiation compared to sham-irradiated rats (Figure 3). The level of CD56^+^ positive cells in heart decreased following kidney irradiation. These findings suggest no increased immune cell presence outside the targeted irradiation volume at this late time point.

### 3.3. Confirmation of the T Cell Knock down in the WAG^CD247-/-^ Rat

**Figure 4:**
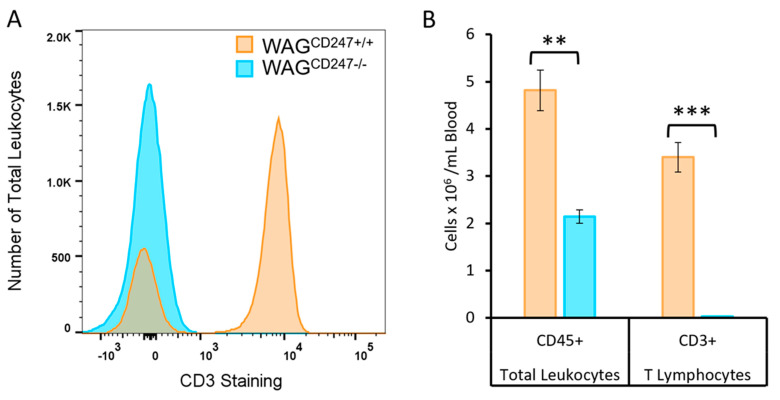
Effective knock down of circulating T lymphocytes in WAG^CD247-/-^ rats. (**A**) Representative histogram of peripheral blood mononuclear cell showing CD3^+^ staining cell counts gated on CD45^+^ total leukocytes demonstrating depletion of CD3^+^ T lymphocytes in circulation of male WAG^CD247-/-^ rats at 5–6 weeks of age. (**B**) Summary of the numbers of CD45+ total leukocytes and CD3^+^ T lymphocytes in circulation of WAG^CD247-/-^ compared to their wild-type WAG littermate controls. CD3^+^ lymphocytes were present at very low levels in WAG^CD247-/-^ rats. Data are mean + SEM, *n* = 3–5 per group. ** = *p* < 0.01 vs. wild-type. *** = *p* < 0.001 vs. wild-type.

To demonstrate the functional role of T lymphocytes in mediating radiation nephropathy and/or cardiac injury, we induced a frameshift deletion in *CD247*, encoding the CD3 zeta chain in the wild-type WAG/RijCmcr rat. Flow cytometry confirmed a >99% depletion in circulating CD3^+^ T lymphocytes in the blood of WAG^CD247-/-^ compared to wild-type rats, which also reduced the number of total white cells to less than half of those in wild-type rats (Figure 4). 

### 3.4. Genetic Knock down of T Cells Decreases Radiation Nephropathy in WAG Rats

**Figure 5:**
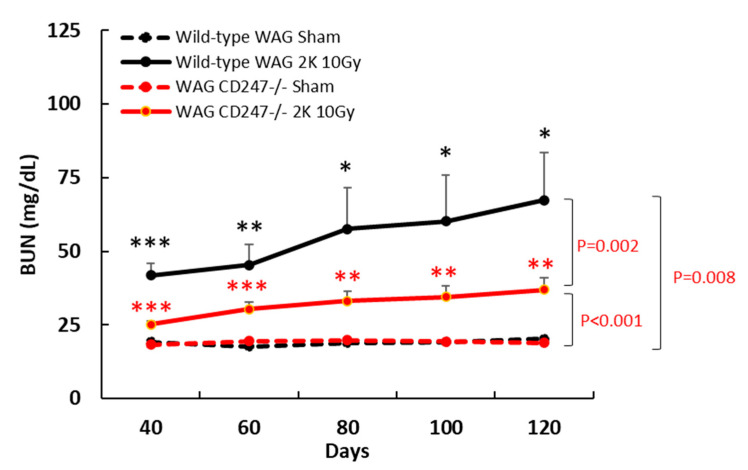
Blood urea nitrogen after local irradiation of the kidneys with 10 Gy of X-rays in wild-type and WAG^CD247-/-^ rats. BUN levels 40–120 days after the start of the study. Data are mean + SEM. *n* = 6/group. * = *p* < 0.05 vs. wild-type. ** = *p* < 0.01 vs. wild-type. *** = *p* < 0.001 vs. wild-type.

Local irradiation of both kidneys in wild-type WAG rats with 10 Gy resulted in elevated BUN levels, a biomarker of kidney dysfunction, after 40 days that remained elevated for at least 120 days compared with the control sham-irradiated rats (*p* = 0.008) (Figure 5). Genetic knock down of T cells in WAG^CD247-/-^ resulted in no difference in BUN levels in sham-irradiated rats compared with wild-type WAG rats that were sham-irradiated (Figure 5). BUN levels were significantly lower in irradiated WAG^CD247-/-^ rats between 40–120 days than in irradiated wild type WAG rats (*p* = 0.002), indicating an important role for T cells in mediating radiation nephropathy (Figure 5). However, an increased level of BUN was still detected in kidney irradiated WAG^CD247-/-^ rats compared with sham irradiated WAG^CD247-/-^ rats 40–120 days (*p* = 0.001), suggesting that other factors contribute to the manifestation of radiation nephropathy (Figure 5). This supports a significant function for T cells in the pathogenesis of renal radiation injury.

### 3.5. Genetic Knock down of T Cells Decreases Pathological Responses in the Non-Targeted Heart following Irradiation of the Kidneys in WAG Rats

**Figure 6:**
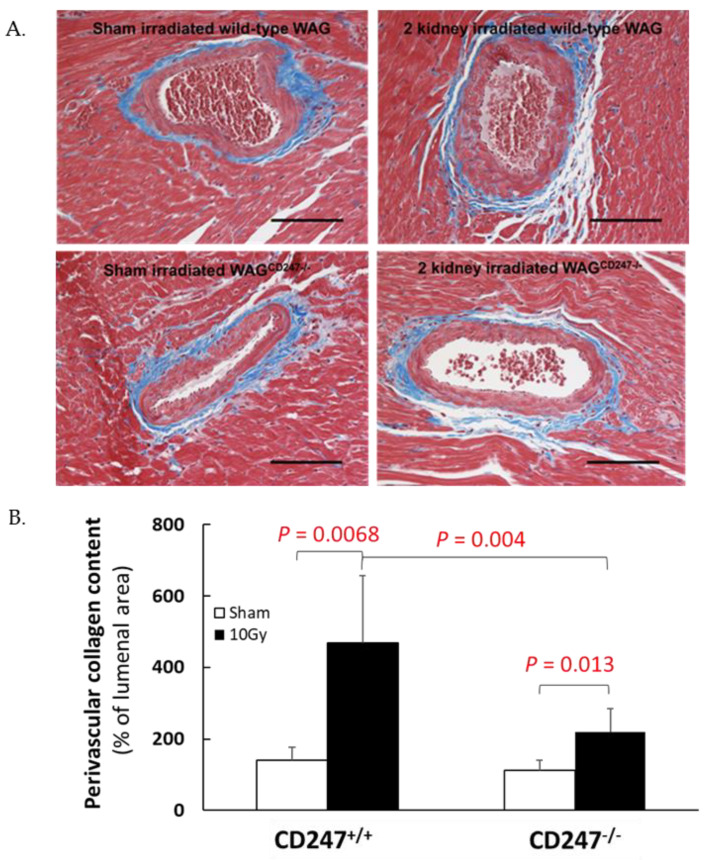
Collagen deposition in heart after local irradiation of kidneys with 10 Gy of X-rays in wild-type and WAG^CD247-/-^ rats. (**A**) Trichrome staining of heart. The horizontal scale bar represents 100 microns. Images are representative data from 3–4 animals per group. (**B**). Quantification of perivascular cardiac collagen content in hearts of wild type and WAG^CD247-/-^ rats 120 days after the start of the study. Data are mean + SD, *n* = 6/group.

Local irradiation of both kidneys in wild-type WAG rats with 10 Gy resulted in elevated perivascular cardiac collagen content at 120 days (Figure 6, *p* = 0.008 vs. sham irradiated controls), confirming our previous observations [4]. In contrast, irradiation of both kidneys in WAG^CD247-/-^ rats caused a lesser increase in perivascular collagen deposition in the heart (Figure 6, *p* = 0.013 vs. sham irradiated controls). The difference in the magnitude of the fibrotic response to 10 Gy in the T cell knock down animals compared with the wild type animals was highly significant (Figure 6, *p* = 0.004), suggesting that during the evolution of the radiation response, T cells supply factors that contribute to the deposition of perivascular collagen in the heart.

### 3.6. Early Radiation Nephropathy Is Not Associated with Engagement of Immune Cells

**Figure 7:**
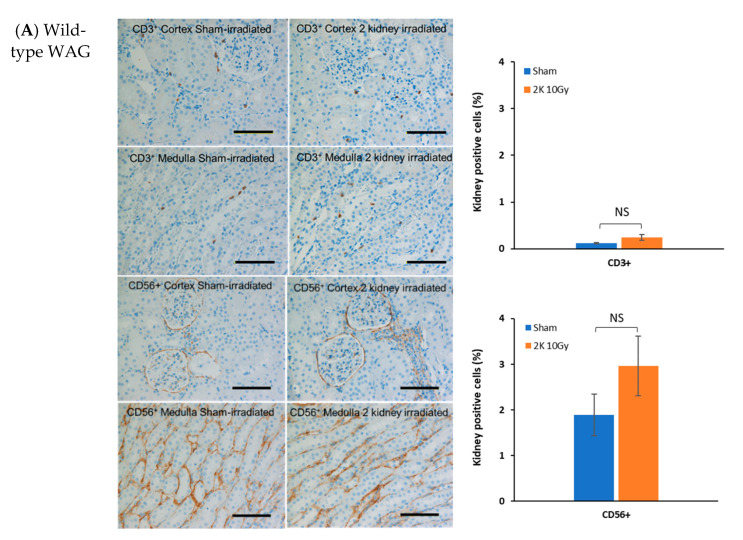

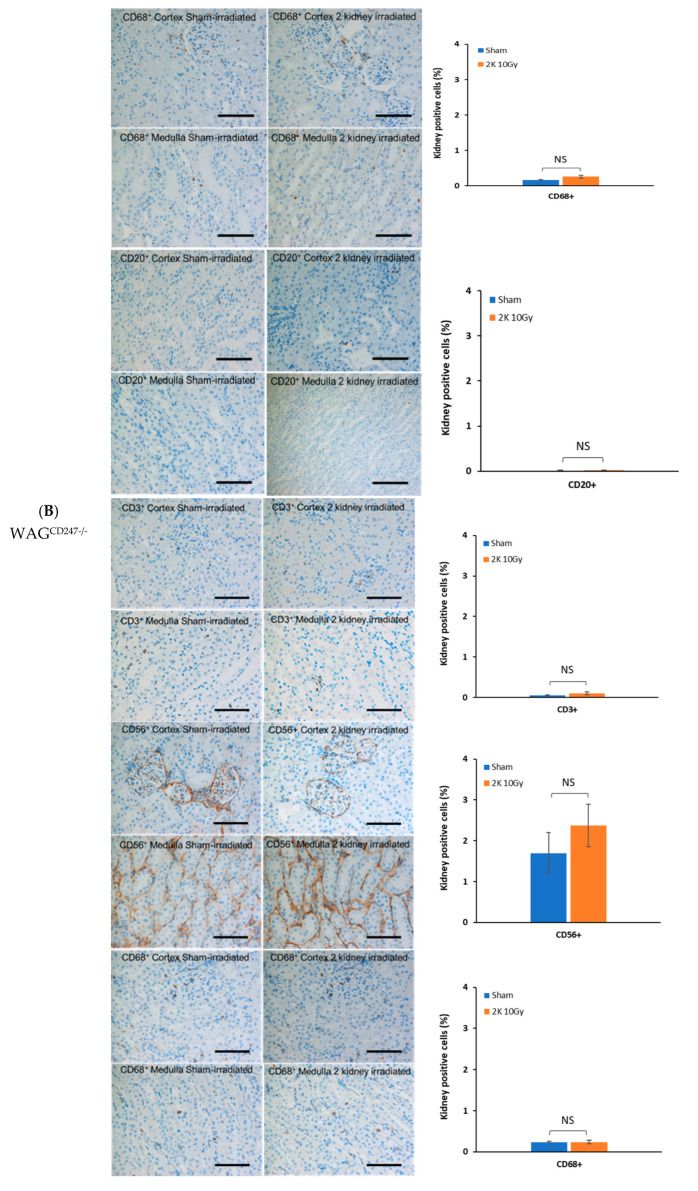

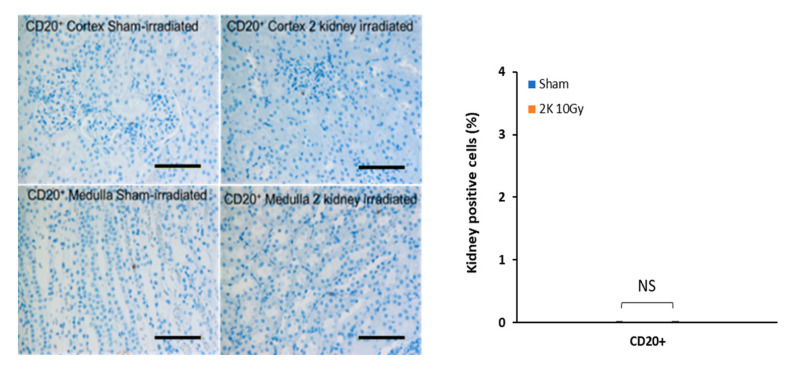
Immune cells in cortex and medulla of kidney 40 days after local irradiation with 10 Gy of X-rays in A. wild-type WAG and B. WAG^CD247-/-^ rats. T cells (CD3^+^), natural killer cells (CD56^+^), macrophages (CD68^+^) and B cells (CD20^+^) appear as brown color. The horizontal scale bar represents 100 microns. Images are representative data from 3–4 animals per group. Quantification of immune cells in kidney. Data are mean + SEM.

To determine whether early radiation nephropathy, evident on day 40 by elevated BUN levels, is linked to engagement of the immune system and the induction of fibrosis, IHC and histology was carried out on kidney tissues from wild-type WAG and WAG^CD247-/-^ rats after sham-irradiation or irradiation with 10 Gy. There was no increase in CD3^+^, CD56^+^, CD68^+^ or CD20^+^ positive cells in the kidneys after irradiation in wild-type WAG rats and WAG^CD247-/-^ rats compared with sham-irradiated controls (Figure 7).

**Figure 8:**
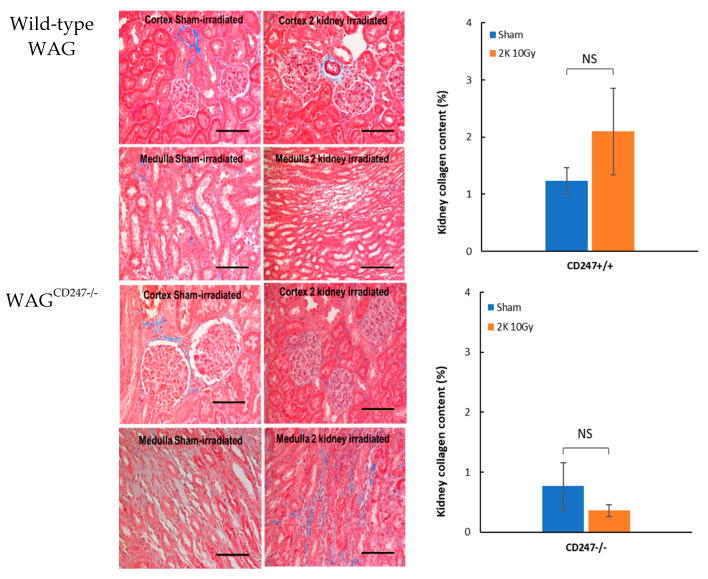
Collagen deposition in kidney after local irradiation with 10 Gy of X-rays in wild type WAG and WAG^CD247-/-^ rats. Trichrome staining of kidney. The horizontal scale bar represents 100 microns. Images are representative data from 3–4 animals per group. Quantification of collagen deposition in kidney. Data are mean + SEM.

Renal collagen content was measured over the area of the entire kidney by trichrome staining and expressed as a percentage of the total area of the kidney section. There was no increase in kidney collagen content after local irradiation with 10 Gy of X-rays in wild-type WAG or WAG^CD247-/-^ rats at 40 days (Figure 8). There was no tubule depletion following local irradiation with 10 Gy of X-rays in wild-type WAG and WAG^CD247-/-^ rats at 40 days (Figure 8). Thus, early radiation nephropathy as indicated by elevated BUN levels on day 40 (Figure 5) is not associated with the engagement of immune cells or induction of fibrosis. 

## 4. Discussion

Our findings show that the engagement of immune cells is part of a late response in radiation-damaged kidneys. It seems to involve several subsets, including CD3^+^, CD56^+^, and CD68^+^ positive cells that accumulate in irradiated kidneys four months after exposure coinciding with organ pathology. There was no accumulation of CD3^+^, CD56^+^, CD68^+^ or CD20^+^ positive cells in the kidney 40 days after irradiation, a time when BUN levels are increasing, but before organ pathology manifest as fibrosis is present. Immune cell accumulation in kidneys is associated with pathological remodeling of the heart outside the radiation field, and in the absence of cardiac immune infiltration indicates that the non-targeted effect on heart might not be directly mediated through cell-to-cell interaction with immune cells. This implies a mechanism that links local radiation injury of kidney to pathology in the non-targeted heart, which would require the transmission of signals from irradiated kidneys to non-irradiated heart. The identity of the systemic signals from the irradiated kidneys remains unclear. The initial response to local radiation may be sensed through conserved damage-associated molecular patterns signaling pathways (DAMPS) that ultimately form a link to the activation of the adaptive arm of the immune system [14,15,16,17].

Using a CRISPR *CD247* gene knock down model in WAG rats, we showed that radiation nephropathy is decreased in the absence of T cells. The involvement of T cells in driving radiation nephropathy is in line with our previous observation in T cell knock down Dahl S rats [4]. The fact that this was seen even without the addition of radiation in that strain underscores a potential role for T cells in driving kidney disease, whether radiation driven or age-related if the genetic predisposition is present [4]. The decrease in natural killer cells and macrophages in sham-irradiated kidneys from 20 days to 120 days may reflect a developmental decline in immune cells [18]. In the present study, we focused on intra-parenchymal immunocytes rather than those in the circulation because the intraparenchymal immune cells are apt to have a closer relation to the mechanisms of parenchymal injury. This local kidney irradiation model avoids the need for bone marrow transplantation and the confounding effect of donor lymphocytes.

Irradiation of the kidneys in T cell knock down WAG^CD247-/-^ rats limited the build-up of perivascular cardiac collagen typically seen in kidney-irradiated wild-type WAG rats [4]. Increased perivascular collagen deposition was not eliminated from kidney-irradiated T cell knock downWAG^CD247-/-^ rats, indicating that cardiac fibrosis is not totally mediated by T cells. Increased perivascular cardiac collagen content is a marker for cardiac fibrosis and a hallmark of radiation-induced heart damage as shown by us [2,4]. In our study using the T cell depleted Dahl S rat, elevated levels of perivascular cardiac collagen already present in sham-irradiated wild-type and T cell depleted Dahl S rats compared with wild-type WAG rats likely obscured any additional effects of radiation [4].

Humans have evolved in an environment continuously exposed to very low levels of background radiation from galactic cosmic rays, terrestrial sources from radionuclides, and intermittent exposure to radiation from solar particle events [19]. Exposure to low levels of ionizing radiation has not resulted in the evolution of unique mechanisms to directly detect exposure. There is no receptor that is specifically engaged by ionizing radiation in mammalian systems to initiate a biological response. The innate immune system functions as the sentinel alerting the body to tissue damage from radiation. Immune cells derived from both lymphoid and myeloid lineages in addition to T cells may be involved in the mechanism underlying non-targeted radiogenic pathology.

In the setting of non-targeted effects, the heart would receive radiation from out-of-field sources; external air scatter originating from the X-ray tube that is independent of distance between anatomical sites and internal low dose X-ray scatter through the rat tissues that is dependent on anatomical distance. Radiation dose will likely be an important determinant in the type and extent of the response as different cellular mechanisms seem to have different thresholds, e.g., micronuclei formation or Nrf2 activation at 20 mGy [20] versus ER stress and proteasome changes at 50 mGy [21] versus ATP release at 100–250 mGy [22,23]. However, direct irradiation of the heart with 10 Gy did not result in pathology [2]. Future studies are needed to define the radiation dose to the kidneys that results in transmission of signals to heart.

Targeted radiation of the tumor is used for cancer treatment. The findings of the current study indicate that the impact of radiation outside the targeted volume needs to be considered in terms of side effects and overall outcomes. Organ systems are interdependent, with signals communicated between each other through the circulation, central nervous and lymphatic systems to maintain homeostasis. Thus, the biological consequences of radiogenic damage inflicted on a localized tumor and the surrounding normal tissue can be communicated to the rest of the body. Our studies confirm that irradiation of a cancer below the diaphragm could cause pathology in the non-targeted heart.

The identity and location of the source within the irradiated volume that communicates signals from cells in injured organs to cells in other organ systems such as the heart is undefined. This non-targeted effect was first measured in 1938 [24], after irradiating the lower third of a rat with 20–40 Gy X-rays. The upper third of the body was shielded to prevent direct actions of radiation on the thymus and spleen. Irradiation of the lower third of the body caused atrophy in the non-targeted and shielded thymus and spleen. In these studies, scatter irradiation may have contributed to the effects observed. This effect was defined as abscopal in 1953 [25] as “at a distance from the irradiated volume but within the same organism”. The findings of the present study indicate that T cells in the immune system are one of the sources of the signals responsible for communicating a signal from the irradiated kidney to the shielded and non-targeted heart. Our previous findings showed that cytokines such as eotaxin, IL-2, IL-13 and IL-18, resulting from targeted irradiation of the kidneys, may transmit signals from irradiated kidneys to the non-targeted heart to initiate pathology [4]. Non-targeted effects represent a paradigm shift from the ‘‘DNA centric’’ view that it is only ionizing radiation that elicits biological effects and subsequent adverse health outcomes solely because of an energy deposition event in the irradiated tissue [3].

Previous studies have shown that macrophages and natural killer cells are the main infiltrating immune cell types in irradiated mouse kidney [26] and irradiated rat kidney [4], respectively. They also represent the more radioresistant fraction of immune cells. Radiation tends to skew the immune balance towards myeloid cells and Tregs, although our model cannot discern which subset is most relevant for driving heart disease. Future studies can test if myeloid cell depletion in our model would break the link between danger-sensing resident innate cells and T cells.

The heart functions primarily as a pump [27] with a secondary function as an endocrine organ [28]. Impairment of heart function can result in symptomatic disease [27]. Radiotherapy increases the occurrence of heart disease in survivors of childhood cancer [29]. These survivors are at risk for harmful cardiovascular outcomes as late as 30 years after fractionated cardiac irradiation with 15 Gy or higher. Direct irradiation of the heart during radiation therapy for breast cancer in adults increases the frequency of major coronary events by 7.4% per Gy with no apparent threshold [30]. The increase in risk for major coronary events is proportional to the dose to the heart, begins within a few years of exposure, and continues for at least 20 years. Understanding the pathogenesis of radiation-induced heart disease is also relevant to setting exposure limits for astronauts in the context of exposure to space radiation during exploratory missions. NASA has designated lifetime radiation exposure limits to minimize or prevent risks of degenerative changes in heart and vasculature. These dose restrictions for the heart are based on direct irradiation of the heart and adjacent arteries [31]. The findings of the current study and others reveal adverse non-targeted effects of radiation on cardiac function [2,32] and structure [4], suggesting radiogenic cardiac pathology can arise from non-cardiac sources in organs below the diaphragm as well as from direct cardiac irradiation. Thus, one or more organs below the diaphragm are the source of a signal that results in disease in the non-targeted heart.

Limitations. The present studies did not show significant immune cell infiltration in kidney 20 days after irradiation, a time when there is cytokine release [4]. Thus, cytokines may have been released from other components of the immune system, as well as non-immune sources such as endothelial cells and fibroblasts during the early response to irradiation of kidneys. We do not know how to link early cytokine release with late pathology in the heart. Future immune profiling investigations are needed to determine the timing and extent of immune cell infiltration in kidney. Studies are needed to examine the role of the large numbers of natural killer cells present in the kidney following irradiation, and in the mechanisms underlying the subsequent cardiac pathology. Our use of a constitutive gene knock down rat allowed us to show T cells play a major role in driving pathology to the non-targeted heart but did not completely explain this effect. This new finding provides the rationale to develop a time-dependent conditional gene knock down rat in which expression of the CD247 gene is temporally controlled to assess mechanisms. Our study focused on normal tissue injury. Further studies are needed to examine whether local irradiation of tumors results in pathology of non-targeted normal tissues such as heart.

## 5. Conclusions

We conclude that engagement of immune cells is a late response to irradiation of the kidneys. The response is specific to individual immune cell types in kidneys. T cells function as effector cells in communicating signals from the irradiated kidneys that cause pathologic remodeling of non-targeted heart. Our findings indicate a novel origin of radiogenic pathology in the heart.

## Data Availability

The data will be deposited in the NASA Life Sciences Data Archive after publication.

## References

[1] Belzile-Dugas E., Eisenberg M.J. (2021). Radiation-Induced Cardiovascular Disease: Review of an Underrecognized Pathology. J. Am. Heart Assoc..

[2] Lenarczyk M., Lam V., Jensen E., Fish B.L., Su J., Koprowski S., Komorowski R.A., Harmann L., Migrino R.Q., Li X.A. (2013). Cardiac injury after 10 gy total body irradiation: Indirect role of effects on abdominal organs. Radiat Res.

[3] Morgan W.F., Sowa M.B. (2015). Non-targeted effects induced by ionizing radiation: Mechanisms and potential impact on radiation induced health effects. Cancer Lett..

[4] Lenarczyk M., Laiakis E.C., Mattson D.L., Johnson B.D., Kronenberg A., North P.E., Komorowski R., Mäder M., Baker J.E. (2020). Irradiation of the kidneys causes pathologic remodeling in the nontargeted heart: A role for the immune system. FASEB BioAdvances.

[5] Stewart B.J., Ferdinand J.R., Young M.D., Mitchell T.J., Loudon K.W., Riding A.M., Richoz N., Frazer G.L., Staniforth J.U., Vieira Braga F.A. (2019). Spatiotemporal immune zonation of the human kidney. Science.

[6] Singh N., Avigan Z.M., Kliegel J.A., Shuch B.M., Montgomery R.R., Moeckel G.W., Cantley L.G. (2019). Development of a 2-dimensional atlas of the human kidney with imaging mass cytometry. JCI Insight.

[7] Rudemiller N., Lund H., Jacob H.J., Geurts A.M., Mattson D.L. (2014). CD247 modulates blood pressure by altering T-lymphocyte infiltration in the kidney. Hypertension.

[8] Baker J.E., Fish B.L., Su J., Haworth S.T., Strande J.L., Komorowski R.A., Migrino R.Q., Doppalapudi A., Harmann L., Allen Li X. (2009). 10 Gy total body irradiation increases risk of coronary sclerosis, degeneration of heart structure and function in a rat model. Int. J. Radiat. Biol..

[9] Abais-Battad J.M., Alsheikh A.J., Pan X., Fehrenbach D.J., Dasinger J.H., Lund H., Roberts M.L., Kriegel A.J., Cowley Jr A.W., Kidambi S. (2019). Dietary effects on Dahl salt-sensitive hypertension, renal damage, and the T lymphocyte transcriptome. Hypertension.

[10] Alsheikh A.J., Lund H., Dasinger J.H., Abais-Battad J.M., Fehrenbach D.J., Mattson D.L. (2019). Renal nerves and leukocyte infiltration in the kidney during salt-sensitive hypertension. Am. J. Physiol. -Regul. Integr. Comp. Physiol..

[11] Ward J., Rehg J. (2014). Rodent immunohistochemistry: Pitfalls and troubleshooting. Vet. Pathol..

[12] Shapiro S.S., Wilk M.B. (1965). An analysis of variance test for normality (complete samples). Biometrika.

[13] Mann H.B., Whitney D.R. (1947). On a test of whether one of two random variables is stochastically larger than the other. Ann. Math. Stat..

[14] McBride W.H., Chiang C.S., Olson J.L., Wang C.C., Hong J.H., Pajonk F., Dougherty G.J., Iwamoto K.S., Pervan M., Liao Y.P. (2004). A sense of danger from radiation. Radiat Res.

[15] Purbey P.K., Scumpia P.O., Kim P.J., Tong A.J., Iwamoto K.S., McBride W.H., Smale S.T. (2017). Defined Sensing Mechanisms and Signaling Pathways Contribute to the Global Inflammatory Gene Expression Output Elicited by Ionizing Radiation. Immunity.

[16] Harding S.M., Benci J.L., Irianto J., Discher D.E., Minn A.J., Greenberg R.A. (2017). Mitotic progression following DNA damage enables pattern recognition within micronuclei. Nature.

[17] Menendez D., Shatz M., Azzam K., Garantziotis S., Fessler M.B., Resnick M.A. (2011). The Toll-like receptor gene family is integrated into human DNA damage and p53 networks. PLoS Genet.

[18] Montecino-Rodriguez E., Berent-Maoz B., Dorshkind K. (2013). Causes, consequences, and reversal of immune system aging. J. Clin. Investig..

[19] Baker J.E., Moulder J.E., Hopewell J.W. (2011). Radiation as a risk factor for cardiovascular disease. Antioxid Redox Signal.

[20] Rodrigues-Moreira S., Moreno S.G., Ghinatti G., Lewandowski D., Hoffschir F., Ferri F., Gallouet A.S., Gay D., Motohashi H., Yamamoto M. (2017). Low-Dose Irradiation Promotes Persistent Oxidative Stress and Decreases Self-Renewal in Hematopoietic Stem Cells. Cell Rep.

[21] Brush J.M., Kim K., Sayre J.W., McBride W.H., Iwamoto K.S. (2009). Imaging of radiation effects on cellular 26S proteasome function in situ. Int. J. Radiat. Biol..

[22] Tsukimoto M., Homma T., Ohshima Y., Kojima S. (2010). Involvement of purinergic signaling in cellular response to gamma radiation. Radiat Res.

[23] Schaue D., Iwamoto K.S., McBride W.H., Wood M.D., Mothersill C.E., Tsakanova G., Cresswell T., Woloschak G.E. (2022). Immune Networks in the Context of Low Dose Ionizing Radiation. Biomarkers of Radiation in the Environment. NATO Science for Peace and Security Series A: Chemistry and Biology.

[24] Segal G., Leblond C. (1938). Reaction d’alarme produite par l’action des rayons X sur l’abdomen chez le rat. Compt Rend Soc De Biol.

[25] Mole R. (1953). Whole body irradiation—Radiobiology or medicine?. Br. J. Radiol..

[26] Scharpfenecker M., Floot B., Russell N.S., Stewart F.A. (2012). The TGF-β co-receptor endoglin regulates macrophage infiltration and cytokine production in the irradiated mouse kidney. Radiother. Oncol..

[27] Libby P., Bonow R.O., Mann D.L., Tomaselli G.F., Bhatt D., Solomon S.D., Braunwald E. (2021). Braunwald’s Heart Disease-E-Book: A Textbook of Cardiovascular Medicine.

[28] Zhao J., Pei L. (2020). Cardiac endocrinology: Heart-derived hormones in physiology and disease. Basic Transl. Sci..

[29] Mulrooney D.A., Yeazel M.W., Kawashima T., Mertens A.C., Mitby P., Stovall M., Donaldson S.S., Green D.M., Sklar C.A., Robison L.L. (2009). Cardiac outcomes in a cohort of adult survivors of childhood and adolescent cancer: Retrospective analysis of the Childhood Cancer Survivor Study cohort. BMJ.

[30] Darby S.C., Ewertz M., McGale P., Bennet A.M., Blom-Goldman U., Brønnum D., Correa C., Cutter D., Gagliardi G., Gigante B. (2013). Risk of ischemic heart disease in women after radiotherapy for breast cancer. N. Engl. J. Med..

[31] Huff J., Cucinotta F.A. (2009). Risk of degenerative tissue or other health effects from radiation exposure. Human Health and performance risks of space exploration missions National Aeronautics and Space Administration, NASA SP-2009-3405. Human Health and Performance Risks of Space Exploration Missions.

[32] Wang Z., Jia Z., Zhou Z., Zhao X., Wang F., Zhang X., Tse G., Li G., Liu Y., Liu T. (2022). Long-Term Cardiac Damage Associated With Abdominal Irradiation in Mice. Front. Pharmacol..

