# Correction: Lenarczyk et al. T Cells Contribute to Pathological Responses in the Non-Targeted Rat Heart following Irradiation of the Kidneys. *Toxics* 2022, *10*, 797

**DOI:** 10.3390/toxics11020183

**Published:** 2023-02-16

**Authors:** Marek Lenarczyk, Ammar J. Alsheikh, Eric P. Cohen, Dörthe Schaue, Amy Kronenberg, Aron Geurts, Slade Klawikowski, David Mattson, John E. Baker

**Affiliations:** 1 Radiation Biosciences, Medical College of Wisconsin, Milwaukee, WI 53226, USA; 2 Department of Physiology, Medical College of Wisconsin, Milwaukee, WI 53226, USA; 3 Department of Medicine, Division of Nephrology, New York University, New York, NY 10016, USA; 4 Department of Radiation Oncology, University of California at Los Angeles, Los Angeles, CA 90095, USA; 5 Biological Systems and Engineering, Lawrence Berkeley National Laboratory, Berkeley, CA 94720, USA; 6 Department of Radiation Oncology, Medical College of Wisconsin, Milwaukee, WI 53226, USA; 7 Department of Physiology, Medical College of Georgia, Augusta, GA 30912, USA

## 1. Error in Figure

In the original publication [1], there was a mistake in Figure 6 as published. “Cardiac fibrosis” should not be shown in Figure 6B. The corrected Figure 6 appears below.

## 2. Figure Legend

In the original publication, there was a mistake in the legend for Figure 7. A part of the text is missing at the beginning of the legend in Figure 7. The correct legend appears below.

**Figure 7.** Immune cells in cortex and medulla of kidney 40 days after local irradiation with 10 Gy of X-rays in A. wild-type WAG and B. WAG^CD247-/-^ rats. T cells (CD3^+^), natural killer cells (CD56^+^), macrophages (CD68^+^) and B cells (CD20^+^) appear as brown color. The horizontal scale bar represents 100 microns. Images are representative data from 3–4 animals per group. Quantification of immune cells in kidney. Data are mean + SEM.

The authors state that the scientific conclusions are unaffected. This correction was approved by the Academic Editor. The original publication has also been updated.

## Figures and Tables

**Figure 6:**
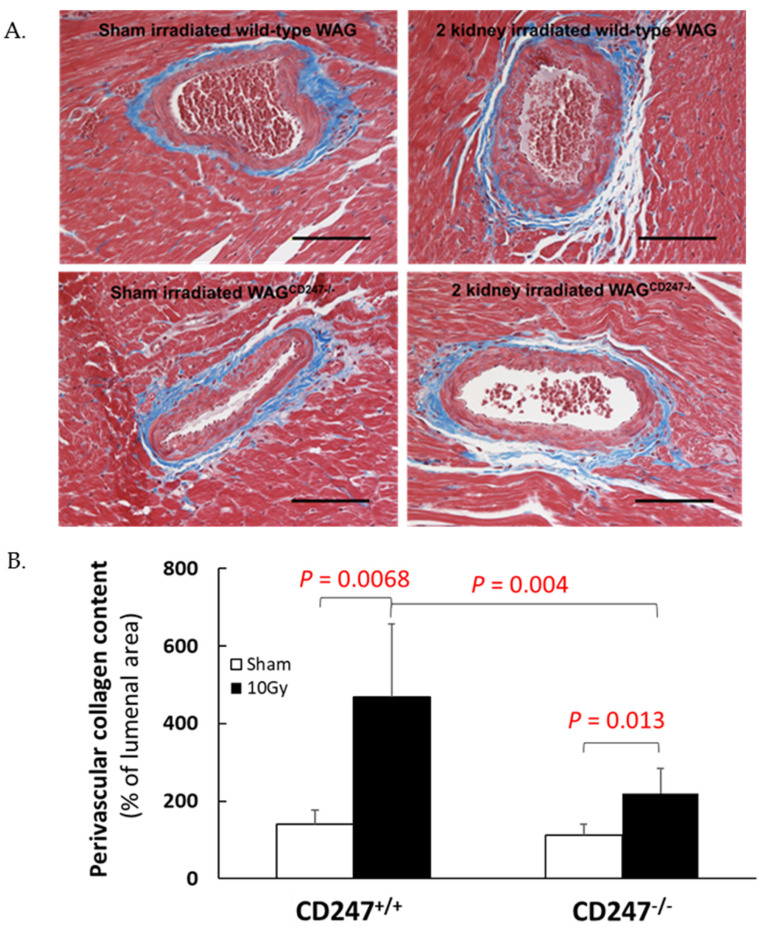
Collagen deposition in heart after local irradiation of kidneys with 10 Gy of X-rays in wild-type and WAG^CD247-/-^ rats. (**A**) Trichrome staining of heart. The horizontal scale bar represents 100 microns. Images are representative data from 3–4 animals per group. (**B**). Quantification of perivascular cardiac collagen content in hearts of wild type and WAG^CD247-/-^ rats 120 days after the start of the study. Data are mean + SD, *n* = 6/group.
